# Bone turnover markers associated with cognitive dysfunction in patients undergoing hemodialysis: a cross-sectional study

**DOI:** 10.1093/jbmrpl/ziag124

**Published:** 2026-08-05

**Authors:** Kazuhiko Kato, Akio Nakashima, Chiharu Aizawa, Arisa Kobayashi, Shunichiro Shinagawa, Ichiro Ohkido, Mitsuyoshi Urashima, Takashi Yokoo

**Affiliations:** Division of Nephrology and Hypertension, Department of Internal Medicine, The Jikei University School of Medicine, Tokyo 105-8461, Japan; Division of Molecular Epidemiology, The Jikei University School of Medicine, Tokyo 105-8461, Japan; Division of Nephrology and Hypertension, Department of Internal Medicine, The Jikei University School of Medicine, Tokyo 105-8461, Japan; Division of Nephrology and Hypertension, Department of Internal Medicine, The Jikei University School of Medicine, Tokyo 105-8461, Japan; Division of Molecular Epidemiology, The Jikei University School of Medicine, Tokyo 105-8461, Japan; Division of Nephrology and Hypertension, Department of Internal Medicine, The Jikei University School of Medicine, Tokyo 105-8461, Japan; Department of Psychiatry, The Jikei University School of Medicine, Tokyo 105-8461, Japan; Division of Nephrology and Hypertension, Department of Internal Medicine, The Jikei University School of Medicine, Tokyo 105-8461, Japan; Division of Molecular Epidemiology, The Jikei University School of Medicine, Tokyo 105-8461, Japan; Division of Nephrology and Hypertension, Department of Internal Medicine, The Jikei University School of Medicine, Tokyo 105-8461, Japan

**Keywords:** cognitive dysfunction, hemodialysis, bone-specific alkaline phosphatase, total alkaline phosphatase, tartrate-resistant acid phosphatase 5b

## Abstract

Cognitive impairment is common in patients undergoing hemodialysis; however, evidence linking routinely measured bone turnover markers to cognition remains limited. In this exploratory cross-sectional study, we aimed to evaluate whether bone-specific alkaline phosphatase (BAP), total alkaline phosphatase (ALP), and tartrate-resistant acid phosphatase 5b (TRACP-5b) are related to global cognitive function in older patients undergoing hemodialysis. Adults aged ≥65 yr receiving maintenance hemodialysis were enrolled at seven centers in Tokyo and Chiba, Japan (November 2021-November 2022). Global cognition was assessed using the Montreal Cognitive Assessment (MoCA) and Mini-Mental State Examination (MMSE). Multivariable linear regression with robust SEs and facility fixed effects was used to examine associations with MoCA scores across sequentially adjusted models. Because of a ceiling effect, MMSE was analyzed using logistic regression with MMSE ≤23 as the outcome (unadjusted and age-/sex-adjusted). For statistical analyses, bone turnover markers were natural log-transformed. Among 380 participants (median age 74 yr; 67.6% male), higher BAP was consistently associated with lower MoCA scores (fully adjusted β −1.03; 95% CI, −1.90 to −0.16). Total ALP showed inverse associations in less-adjusted models; however, these associations attenuated after full adjustment (β −.88; 95% CI, −1.89 to 0.14). TRACP-5b was not clearly associated with MoCA scores. Higher BAP and total ALP were associated with higher odds of MMSE-defined cognitive dysfunction, whereas TRACP-5b was not. These findings suggest a potential association between bone formation-related markers and cognitive performance in older patients undergoing hemodialysis. Prospective and mechanistic studies are warranted to clarify causality and clinical utility.

## Graphical Abstract

**Graphical Abstract:**
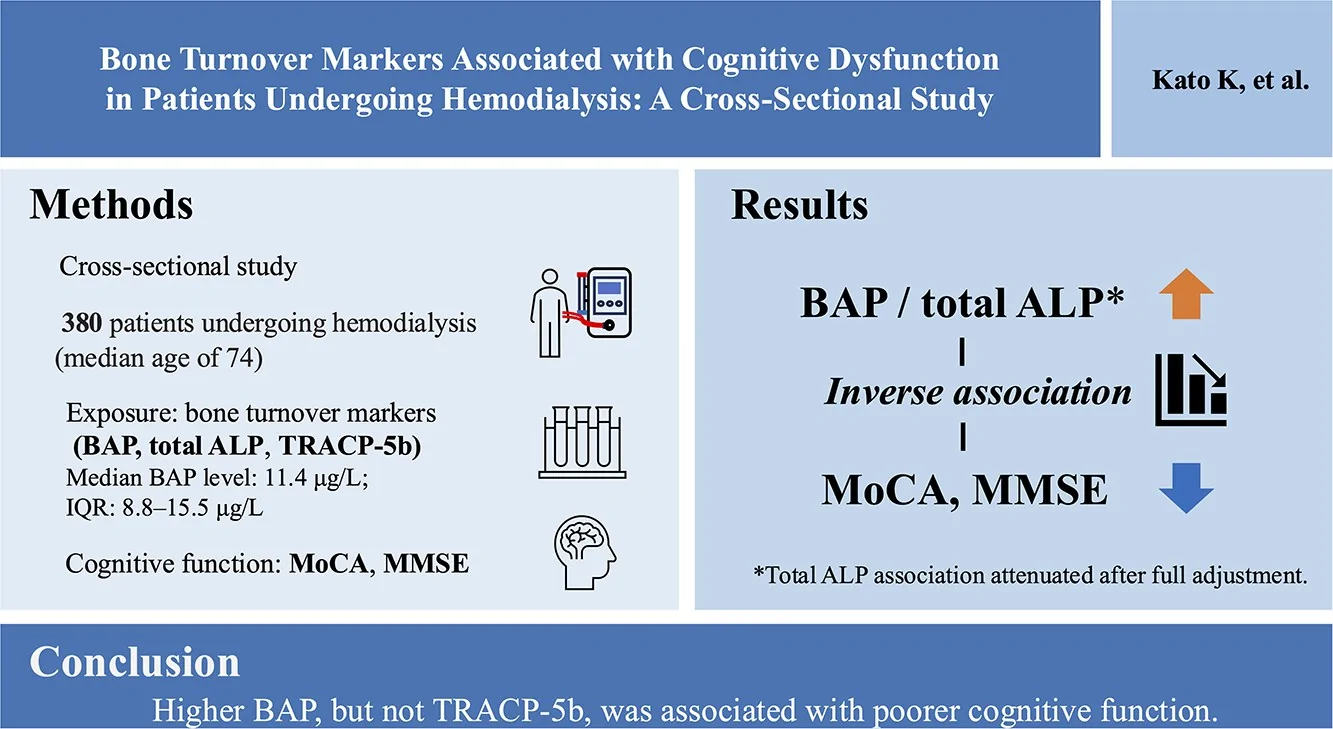

## Introduction

Cognitive impairment is common in patients receiving maintenance hemodialysis^1^ and is associated with clinically important consequences, such as diminished quality of life, reduced treatment adherence, and higher mortality.^2^ The prevalence of cognitive impairment in patients undergoing hemodialysis has been estimated to be roughly twice that observed in the general population.^3^ Several biological and clinical pathways have been proposed, including the amyloid cascade hypothesis, cerebrovascular injury, chronic inflammation, uremic toxins, oxidative stress, and dialysis-related hemodynamic instability. Overall, cognitive impairment in patients undergoing hemodialysis is generally understood as a multifactorial condition, and its pathophysiology remains incompletely defined.^4^

CKD–mineral and bone disorder (CKD–MBD) has been reported to be associated with cognitive dysfunction in patients undergoing hemodialysis.^5^^,^^6^ Beyond its traditional manifestations in bone disease and cardiovascular complications, CKD–MBD is also recognized as a systemic phenotype that overlaps with other CKD-related pathologies, including anemia, chronic inflammation, and immune dysregulation. CKD–MBD may also contribute to functional decline, such as sarcopenia and cognitive impairment.^7–9^ In addition, elevated bone turnover markers have been associated with adverse outcomes in patients with CKD and those undergoing hemodialysis.^10–17^ However, direct evidence of an association between these markers and cognitive function in patients undergoing hemodialysis remains limited.

In non-dialysis settings, several observational studies have reported that higher total alkaline phosphatase (ALP) levels are associated with poorer cognitive performance and cognitive impairment across diverse clinical and community-based populations.^18–23^ Mechanistically, tissue-nonspecific ALP has been implicated in neurodegeneration, including experimental evidence linking it to extracellular tau-mediated neurotoxicity.^24^ However, despite growing interest in bone-related biomarkers and cognition in patients undergoing hemodialysis,^25^ studies specifically reporting the evaluation of routinely measured bone turnover markers, such as total ALP, bone-specific ALP (BAP), and tartrate-resistant acid phosphatase 5b (TRACP-5b), in relation to cognitive function remain limited.

In this exploratory cross-sectional study, we aimed to examine whether bone turnover, as reflected by routinely measured bone turnover markers, is associated with global cognitive function in older patients undergoing hemodialysis. We focused on total ALP, BAP, and TRACP-5b, and we evaluated cognitive performance using the Montreal Cognitive Assessment (MoCA) and Mini-Mental State Examination (MMSE).

## Materials and methods

### Ethical approval

All procedures involving human participants were conducted in accordance with the ethical standards of the institutional and national research committees, as well as the 1964 Declaration of Helsinki and its later amendments or comparable ethical standards. Ethical approval was obtained from the IRB of the Jikei University School of Medicine (Approval No. 33-183(10800)). Written informed consent was obtained from all participants before inclusion.

### Study population

This exploratory cross-sectional study included adults aged ≥65 yr undergoing maintenance hemodialysis. Patients were eligible if they had no previous diagnosis of dementia and were not receiving dementia-specific treatment.

We excluded individuals with acute delirium, conditions that would preclude valid cognitive assessment (eg, major neurologic or psychiatric disorders), or acute medical problems at the time of assessment, including severe electrolyte abnormalities or systemic illnesses requiring treatment (eg, pneumonia, acute heart failure, or active infection). Patients hospitalized for any acute medical condition within the preceding 3 mo were also excluded. Consistent with the study design, patients were also excluded if they had been on hemodialysis for <3 mo, received <3 hemodialysis sessions per week, or lacked serum bone turnover marker data.

To reduce potential selection bias, patients were recruited consecutively from seven dialysis centers in Chiba and Tokyo, Japan, between November 2021 and November 2022. We used the same cohort and cognitive testing protocol that were used in previously published work,^5^ but we examined different exposures and research questions.

### Clinical and laboratory data collection

Baseline demographic information, comorbidities, and concomitant medications were extracted from medical records at enrollment. History of stroke was defined as a history of cerebral infarction or cerebral hemorrhage. Routine laboratory tests were obtained before the first hemodialysis session of the week using standard clinical assays, including blood urea nitrogen (mg/dL), serum albumin (g/dL), hemoglobin (g/dL), C-reactive protein (mg/dL), γ-glutamyl transferase (U/L), calcium (mg/dL), phosphate (mg/dL), intact parathyroid hormone (pg/mL), and total ALP (U/L; International Federation of Clinical Chemistry and Laboratory Medicine-standardized method). Dialysis adequacy was assessed using single-pool Kt/V. Blood samples were centrifuged for 10 min, aliquoted, and stored at −80 °C until analysis. Serum 25-hydroxyvitamin D (25OHD; ng/mL), BAP (μg/L), and TRACP-5b (mU/dL) were measured at SRL Inc. (Tokyo, Japan) using established immunoassays: electrochemiluminescence, chemiluminescent enzyme, and enzyme immunoassays, respectively. Serum intact fibroblast growth factor 23 (FGF23; pg/mL) and soluble α-klotho (pg/mL) were measured using commercially available ELISA kits, as previously reported.^5^ Educational level and smoking history were additionally collected using a structured questionnaire.

### Cognitive function assessment

Global cognition was evaluated using the validated Japanese versions of the MoCA^26^^,^^27^ and MMSE.^28^ To reduce inter-rater variability, all assessments were administered by a single trained investigator. Cognitive testing was conducted during hemodialysis with participants either seated or supine and was deferred in cases of intradialytic hypotension.

### Exposure and outcome

The primary exposures were serum bone turnover markers (BAP, total ALP, and TRACP-5b). The primary outcome was global cognitive function assessed using MoCA and MMSE scores.

### Statistical analysis

Continuous variables are presented as mean ± SD for approximately normally distributed data and as median (interquartile range [IQR]) for skewed data. Variables with skewed distributions, including BAP, total ALP, TRACP-5b, dialysis vintage, C-reactive protein, γ-glutamyl transferase, intact parathyroid hormone, 25OHD, FGF23, and soluble α-klotho, were natural log-transformed. We examined the correlation between BAP and total ALP using Spearman's rank correlation coefficient.

Multivariable linear regression analyses with robust SEs were performed to examine the associations between serum bone turnover markers and MoCA scores. Covariates were selected using the disjunctive cause criterion, whereby potential confounders were identified based on prior literature as variables causally related to the exposure, the outcome, or both.^29^ Facility was accounted for using fixed effects, and smoking status was modeled as a three-level categorical variable (no/yes/unknown).

Model 1 was adjusted for age, sex, and facility. Model 2 additionally adjusted for smoking status and education level (≤12 yr). Model 3 further adjusted for dialysis vintage, pre-dialysis systolic blood pressure, diabetes mellitus, cardiovascular disease, history of stroke, chronic hepatitis, and hepatocellular carcinoma. Model 4 further adjusted for CKD–MBD-related medications (phosphate binders, vitamin D receptor activators, calcimimetics, denosumab, and systemic glucocorticoids). Model 5 further adjusted for dialysis-related, nutritional, anemia-related, inflammatory, hepatobiliary, and CKD–MBD laboratory parameters, including blood urea nitrogen, single-pool Kt/V, serum albumin, BMI, hemoglobin, C-reactive protein, γ-glutamyl transferase, calcium, phosphate, intact parathyroid hormone, 25OHD, FGF23, and soluble α-klotho. Multicollinearity was assessed using variance inflation factors, and no substantial multicollinearity was detected.

To explore potential non-linear associations between bone turnover markers and cognitive function, restricted cubic spline analyses were performed for BAP, total ALP, and TRACP-5b, with the MoCA score treated as a continuous outcome. These models were fitted using three knots and adjusted for the same covariates included in the fully adjusted multivariable linear regression model (Model 5).

Additionally, because the distribution of MMSE scores showed a marked ceiling effect, linear regression using MMSE as a continuous outcome was considered inappropriate. Therefore, we conducted logistic regression analyses defining cognitive dysfunction as MMSE ≤ 23. To avoid overfitting due to the limited number of outcome events, we fitted only an unadjusted model and an age- and sex-adjusted model.

As sensitivity analyses, logistic regression models were fitted using dichotomized MoCA outcomes. Cognitive dysfunction was defined using the original MoCA cutoff (≤25) and a lower cutoff (≤22), which has been reported to yield better sensitivity and specificity than the original threshold in prior studies.^30^ For each cutoff, we report an unadjusted model and an age-, sex-, and facility-adjusted model (facility fixed effects).

As an additional exploratory analysis, we examined whether the associations between bone turnover markers and global MoCA scores were driven by specific cognitive domains. MoCA domain scores, including visuospatial/executive function, naming, attention, language, abstraction, delayed recall, and orientation, were analyzed as continuous outcomes. Separate linear regression models with robust SEs were fitted for each bone turnover marker. We present an unadjusted model and Model 1, which is adjusted for age, sex, and facility. These domain-level analyses were exploratory. We did not perform analogous domain-level regression analyses for the MMSE because the total MMSE score showed a marked ceiling effect in this cohort, and individual MMSE domains have even more restricted score ranges. We therefore considered MMSE domain-level regression analyses to be statistically unstable and potentially misleading.

To further contextualize the magnitude of the association between bone turnover markers and cognitive function, we performed additional exploratory analyses to evaluate the clinical determinants of MoCA score. Univariable linear regression models with robust SEs were fitted for each clinical variable. We then fitted a fully adjusted multivariable linear regression model including BAP and the same covariates included in the primary fully adjusted model. Standardized β coefficients were obtained from the fully adjusted model to compare the relative magnitude of associations across clinical variables. We focused these determinant analyses on MoCA because MoCA was analyzed as a continuous outcome in the main analyses. We did not perform analogous comprehensive multivariable determinant analyses for MMSE because MMSE scores showed a marked ceiling effect and the number of participants with MMSE-defined cognitive dysfunction was limited, making such analyses potentially unstable and prone to overfitting.

All statistical tests were two-sided, and *p* values <.05 were considered statistically significant. Except for smoking status (8% missing), missingness was <5% for all variables; therefore, complete-case analyses were conducted, treating missing smoking status as an “unknown” category to retain the analytic sample. Given the exploratory nature of the study, no adjustment for multiple comparisons was applied, and no formal sample size calculation was conducted. All statistical analyses were performed using Stata/BE, version 18.0 (StataCorp LLC, College Station, TX, USA).

## Results

### Study population

Of the 403 consecutively enrolled patients aged ≥65 yr receiving maintenance hemodialysis, 10 with acute medical conditions and 12 without available serum bone turnover marker measurements were excluded. One additional patient was excluded for a protocol-defined reason (receiving fewer than three hemodialysis sessions per week). Thus, 380 patients were included in the final analyses.

### *Patient characteristics* (Table 1)

**Table 1:** Patient characteristics (*n* = 380).

Variable	Value		Missing, *n* (%)
**Age, years**	74	(70-80)	0 (0%)
**Sex (male), *n* (%)**	257	(67.6)	0 (0%)
**Smoking status, *n* (%)**			0 (0%)
**Yes**	227	(59.7)	
**No**	124	(32.6)	
**Unknown**	29	(7.6)	
**Education years ≤12 yr, *n* (%)**	224	(59.4)	3 (0.7%)
**Comorbidities**			
** Dialysis vintage, months**	86	(35-167)	0 (0%)
** Pre-dialysis systolic blood pressure, mmHg**	146	±23	0 (0%)
** Diabetes mellitus, *n* (%)**	162	(42.6)	0 (0%)
** Cardiovascular disease, *n* (%)**	131	(34.5)	0 (0%)
** History of stroke, *n* (%)**	56	(14.7)	0 (0%)
** Chronic hepatitis, *n* (%)**	14	(3.7)	0 (0%)
** Hepatocellular carcinoma, *n* (%)**	5	(1.3)	0 (0%)
**Treatment**			
** Phosphate binder, *n* (%)**	248	(65.3)	0 (0%)
** Vitamin D receptor activators, *n* (%)**	283	(74.5)	0 (0%)
** Calcimimetics, *n* (%)**	204	(53.7)	0 (0%)
** Denosumab, *n* (%)**	15	(4.0)	0 (0%)
** Systemic glucocorticoid, *n* (%)**	11	(2.9)	0 (0%)
**Laboratory measurements**			
** Urea nitrogen, mg/dL**	62.5	±13.8	0 (0%)
** Single-pool urea clearance (Kt/V)**	1.5	±0.3	18 (4.7%)
** Albumin, g/dL**	3.6	±0.3	0 (0%)
**BMI, kg/m** ^**2**^	21.6	(19.6-24.3)	0 (0%)
** Hemoglobin, g/dL**	10.9	±1.1	0 (0%)
** C-reactive protein, mg/dL**	0.09	(0.03-0.3)	0 (0%)
** γ-glutamyl transferase, U/L**	17	(12-26)	1 (0.3%)
** Calcium, mg/dL**	8.3	(8.0-8.7)	0 (0%)
** Phosphate, mg/dL**	5.2	(4.4-5.9)	0 (0%)
** Intact parathyroid hormone, pg/mL**	158	(94-241)	0 (0%)
** 25OHD, ng/mL**	14.1	(10-19.6)	0 (0%)
** Intact FGF23, pg/mL**	1925	(620-4772)	0 (0%)
** Soluble α-klotho, pg/mL**	381	(299-516)	0 (0%)
** Bone-specific alkaline phosphatase, μg/L**	11.4	(8.8-15.5)	0 (0%)
** Total alkaline phosphatase, U/L**	71	(58-88.5)	0 (0%)
** Tartrate-resistant acid phosphatase 5b, mU/dL**	280	(166-444)	1 (0.3%)
**Cognitive function**			
** Montreal Cognitive Assessment**	25	(22-26)	0 (0%)
** Mini-Mental State Examination**	28	(26-29)	0 (0%)
** Mini-Mental State Examination score ≤ 23, *n* (%)**	39	(10.3)	0 (0%)

Continuous variables are presented as means (± SDs) or medians (interquartile ranges). Categorical variables are expressed as numbers of patients (%).

History of stroke was defined as a history of cerebral infarction or cerebral hemorrhage.

The median age was 74 (IQR 70-80) yr, and 67.6% of the patients were male. The median dialysis vintage was 86 mo. Diabetes mellitus was present in 42.6% of the patients, cardiovascular disease in 34.5%, and a history of stroke in 14.7%. The median BAP, total ALP, and TRACP-5b levels were 11.4 (IQR 8.8-15.5) μg/L, 71 (IQR 58-88.5) U/L, and 280 (IQR 166-444) mU/dL, respectively. The median MoCA score was 25 (IQR 22-26), and the median MMSE score was 28 (IQR 26-29); 10.3% of the participants met the criterion for cognitive dysfunction, defined as an MMSE score ≤ 23. BAP and total ALP were moderately to strongly correlated (Spearman’s ρ = 0.673).

### Association between bone turnover markers and MoCA score

In multivariable linear regression analyses (Table 2), higher log-transformed BAP levels were consistently associated with lower MoCA scores across sequential models. The association was borderline in the unadjusted model (β −.79; 95% CI, −1.58 to −0.001; *p* = .050) and remained statistically significant after full adjustment (Model 5: β −1.03; 95% CI, −1.90 to −0.16; *p* = .021). Higher log-transformed total ALP levels were also inversely associated with the MoCA score in less-adjusted models, but the association attenuated and was no longer significant in the fully adjusted model (Model 5: β −.88; 95% CI, −1.89 to 0.14; *p* = .090). Log-transformed TRACP-5b was not significantly associated with the MoCA score in the unadjusted model or in adjusted models.

**Table 2:** Multivariable linear regression analysis of the association between bone turnover markers and Montreal Cognitive Assessment scores.

	β (95% CI)	*p* value
**Log BAP**		
** Unadjusted**	−.79 (−1.58 to −0.001)	.050
** Model 1**	−.97 (−1.70 to −0.24)	.010
** Model 2**	−.97 (−1.72 to −0.22)	.011
** Model 3**	−.94 (−1.68 to −0.19)	.014
** Model 4**	−.94 (−1.72 to −0.16)	.019
** Model 5**	−1.03 (−1.90 to −0.16)	.021
**Log total ALP**		
** Unadjusted**	−1.18 (−2.04 to −0.32)	.007
** Model 1**	−1.11 (−1.92 to −0.31)	.007
** Model 2**	−.97 (−1.77 to −0.16)	.019
** Model 3**	−.99 (−1.82 to −0.16)	.019
** Model 4**	−.94 (−1.78 to −0.10)	.028
** Model 5**	−.88 (−1.89 to 0.14)	.090
**Log TRACP-5b**		
** Unadjusted**	−.25 (−0.76 to 0.26)	.331
** Model 1**	−.47 (−0.97 to 0.03)	.069
** Model 2**	−.46 (−0.98 to 0.05)	.078
** Model 3**	−.46 (−0.97 to 0.06)	.080
** Model 4**	−.45 (−1.00 to 0.10)	.114
** Model 5**	−.57 (−1.20 to 0.07)	.079

Abbreviations: CI, confidence interval; BAP, bone-specific alkaline phosphatase; ALP, alkaline phosphatase; TRACP-5b, tartrate-resistant acid phosphatase 5b.

Bone turnover markers were natural log–transformed. Facility was adjusted for using fixed effects (indicator variables). Smoking status was modeled as a three-level categorical variable (no/yes/unknown).

**Unadjusted:** Marker only.

**Model 1:** Adjusted for age, sex, and facility.

**Model 2:** Model 1, additionally adjusted for smoking status and education (≤12 yr).

**Model 3:** Model 2, additionally adjusted for dialysis vintage, pre-dialysis systolic blood pressure, diabetes mellitus, cardiovascular disease, history of stroke, chronic hepatitis, and hepatocellular carcinoma.

**Model 4:** Model 3, further adjusted for phosphate binder use, vitamin D receptor activator use, calcimimetics use, denosumab use, and systemic glucocorticoid use.

**Model 5:** Model 4, further adjusted for urea nitrogen, dialysis adequacy (Kt/V), serum albumin, BMI, hemoglobin, C-reactive protein, γ-glutamyl transferase, calcium, phosphate, intact parathyroid hormone, 25OHD, FGF23, and soluble α-klotho.

**Figure 1:**
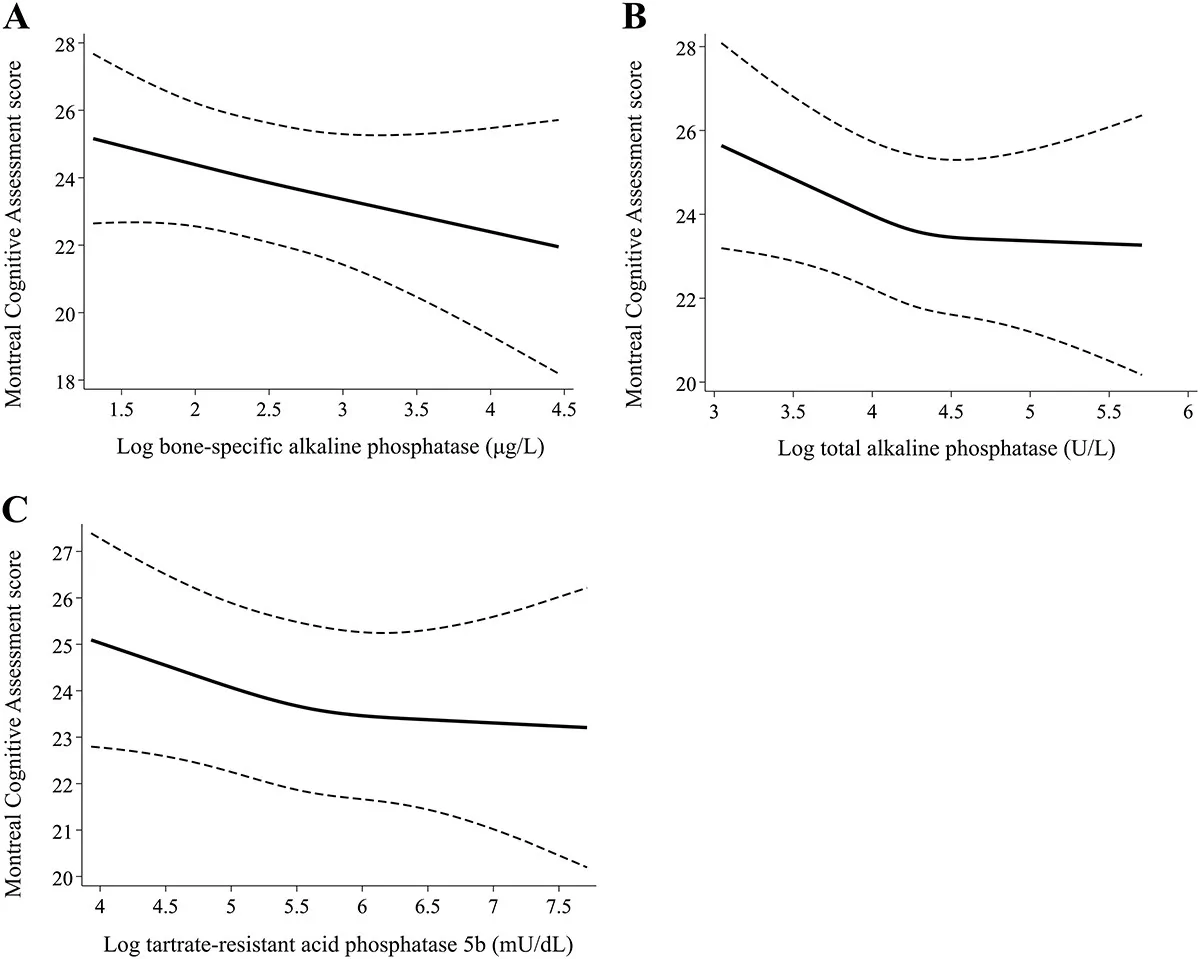
Restricted cubic spline analyses of the associations between bone turnover markers and Montreal Cognitive Assessment scores. (A) Bone-specific alkaline phosphatase, (B) total alkaline phosphatase, and (C) tartrate-resistant acid phosphatase 5b. Solid lines indicate adjusted estimates, and dashed lines indicate 95% CIs. Bone turnover markers were natural log-transformed. Models were fitted with three knots and adjusted for the same covariates as in the fully adjusted multivariable linear regression model (Model 5).

Restricted cubic spline analyses (Figure 1) suggested an approximately linear inverse association of log-transformed BAP and total ALP with the MoCA score; for TRACP-5b, the curve appeared largely flat, without evidence of marked non-linearity.

### *Association with cognitive dysfunction defined by MMSE score ≤ 23* (Table 3)

**Table 3:** Multivariable logistic regression analyses of the association between bone turnover markers and cognitive dysfunction (Mini-Mental State Examination ≤23).

	Odds ratio (95% CI)	*p* value
**Log BAP**		
** Unadjusted**	2.24 (1.25-4.01)	.007
** Age- and sex-adjusted**	2.40 (1.34-4.32)	.003
**Log total ALP**		
** Unadjusted**	3.30 (1.62-6.72)	.001
** Age- and sex-adjusted**	3.56 (1.68-7.52)	.001
**Log TRACP-5b**		
** Unadjusted**	1.31 (0.88-1.97)	.187
** Age- and sex-adjusted**	1.24 (0.82-1.89)	.303

Abbreviations: CI, confidence interval; BAP, bone-specific alkaline phosphatase; ALP, alkaline phosphatase; TRACP-5b, tartrate-resistant acid phosphatase 5b.

Bone turnover markers were natural log–transformed.

**Unadjusted:** marker only.

**Age- and sex-adjusted:** Adjusted for age and sex.

In logistic regression analyses defining cognitive dysfunction as an MMSE score ≤ 23, higher log-transformed BAP and total ALP levels were associated with higher odds of cognitive dysfunction in both the unadjusted and age- and sex-adjusted models (BAP age- and sex-adjusted OR 2.40; 95% CI, 1.34-4.32; *p* = .003; total ALP age- and sex-adjusted OR 3.56; 95% CI, 1.68-7.52; *p* = .001). In contrast, log-transformed TRACP-5b was not associated with MMSE-defined cognitive dysfunction.

### *Sensitivity analyses defining cognitive dysfunction by MoCA score (≤25 and ≤ 22)* (Table S1)

When cognitive dysfunction was defined using the original MoCA cutoff (≤25), none of the bone turnover markers were significantly associated with the outcome in the unadjusted or adjusted models. Using a lower MoCA cutoff (≤22), higher log-transformed BAP and total ALP levels were associated with higher odds of cognitive dysfunction after adjustment (Model 1 OR 1.93; 95% CI, 1.13-3.29; *p* = .015 for BAP; Model 1 OR 2.39; 95% CI, 1.33-4.27; *p* = .003 for total ALP), whereas TRACP-5b remained non-significant.

### *Exploratory domain-level analyses of MoCA scores* (Table S2)

BAP and total ALP showed inverse associations with several MoCA domain scores, including language and abstraction. However, the magnitudes of these associations were small, and the associations were not clearly confined to a single cognitive domain. No clear associations were observed for attention or delayed recall. TRACP-5b did not show a consistent domain-specific pattern.

### *Clinical determinants of MoCA score* (Table S3)

In the fully adjusted model, older age and a history of stroke were independently associated with lower MoCA scores. Log-transformed BAP also remained inversely associated with MoCA score, although its standardized β coefficient was smaller than those for age and history of stroke. Log-transformed intact parathyroid hormone was not clearly associated with MoCA score.

## Discussion

In this exploratory cross-sectional study of older patients undergoing hemodialysis, higher BAP levels were consistently associated with poorer global cognitive performance assessed using the MoCA. Total ALP showed an inverse association with the MoCA score in less-adjusted models, but this association attenuated after full adjustment. However, TRACP-5b was not clearly associated with the MoCA score and appeared largely flat in spline analyses. Because MMSE scores exhibited a marked ceiling effect, MMSE was not analyzed as a continuous outcome. Instead, logistic regression using an MMSE cutoff (≤23) showed higher odds of cognitive dysfunction with higher BAP and total ALP levels but not with TRACP-5b. In sensitivity analyses using dichotomized MoCA outcomes, associations were not observed with the original cutoff (≤25) but emerged with a lower cutoff (≤22). This finding is consistent with information loss when continuous cognitive measures are dichotomized.

The marker-specific pattern suggests that the observed associations may not simply reflect a uniform high-turnover bone state. Specifically, the formation-related markers BAP and total ALP were associated with worse cognition, whereas the resorption-related marker TRACP-5b was not. The lack of association for TRACP-5b may indicate that osteoclast-driven resorption alone is unlikely to explain the observed findings. In additional exploratory analyses, intact parathyroid hormone was not clearly associated with MoCA score, suggesting that the association between BAP and cognitive function was not simply explained by circulating parathyroid hormone levels. BAP and total ALP were moderately to strongly correlated in this cohort, suggesting that total ALP partly reflects bone formation-related activity. However, this correlation was incomplete, and total ALP also includes non-bone-derived ALP activity. The more consistent association of BAP with MoCA score, together with attenuation of the association for total ALP after full adjustment, may suggest that the bone formation-related component captured by BAP is more relevant to cognitive performance than total ALP as a composite marker. Conversely, non-bone-derived ALP may still contribute through extra-skeletal pathways. Because ALP isoenzymes were not measured, the relative contributions of bone-derived and non-bone-derived ALP could not be distinguished in the present study.

Several non-mutually exclusive mechanisms may underlie these associations. One possibility is vascular and ectopic calcification biology. Total ALP largely reflects tissue-nonspecific ALP activity, which may promote calcification by hydrolyzing extracellular pyrophosphate, a key inhibitor of mineralization.^31–33^ A second possibility is a neurodegenerative pathway: experimental work has linked tissue-nonspecific ALP to extracellular tau-mediated neurotoxicity,^24^ and plasma tissue-nonspecific ALP activity has also been reported to differ in patients with mild cognitive impairment and Alzheimer's disease.^34^ A third possibility is systemic illness burden, as BAP and total ALP may reflect chronic inflammation and malnutrition,^35^ both of which are associated with cognitive dysfunction in patients undergoing hemodialysis.^36–38^

To our knowledge, evidence directly linking routinely measured bone turnover markers to cognitive function in patients undergoing hemodialysis remains limited. In non-dialysis settings, higher total ALP has been associated with poorer cognitive performance and cognitive impairment across several observational studies.^18–23^ These findings are directionally consistent with our results for total ALP. In patients undergoing hemodialysis, prior work has examined selected bone-related biomarkers in relation to cognition (eg, receptor activator of nuclear factor kappa-B ligand),^25^ and total ALP has been included as a candidate covariate in some studies.^39^^,^^40^ However, routinely measured bone turnover markers, such as total ALP, BAP, and TRACP-5b, have not been comprehensively evaluated in this context. Accordingly, in the present study, we extend prior work by focusing on routinely available bone turnover markers and comparing formation- and resorption-related markers within the same hemodialysis cohort.

The magnitude of the effect estimates was modest and should be interpreted with caution. Even after extensive adjustment for potential confounders, these markers alone are unlikely to account for the substantially elevated burden of cognitive dysfunction in patients undergoing hemodialysis. In additional analyses of clinical determinants, the standardized β coefficient for BAP with MoCA score was smaller than those for established determinants such as age and history of stroke. Therefore, the observed associations may not be clinically decisive at the individual patient level. Consistently, exploratory MoCA domain-level analyses did not suggest that the associations were driven by a specific cognitive domain, and the domain-level effect estimates were small. Cognitive impairment in patients undergoing hemodialysis is inherently multifactorial. Thus, our findings should be viewed as supporting the hypothesis that these markers represent one component within a broader causal network. Residual confounding cannot be excluded, and BAP and total ALP may partly reflect intermediate processes related to overall systemic health status rather than direct causal effects on cognition. In particular, malnutrition or protein-energy wasting may be associated with both higher bone turnover marker levels and poorer cognition, potentially confounding the observed relationships. Although we adjusted for nutritional indicators such as serum albumin and BMI, these measures may not fully capture malnutrition, protein-energy wasting, or frailty in patients undergoing hemodialysis.

An important limitation of the present study is that cognitive assessments were performed during hemodialysis sessions. Hemodialysis can acutely influence cognitive performance through reductions in cerebral blood flow, blood pressure fluctuations, osmotic shifts, hemodynamic instability, and fatigue during the dialysis session.^41^^,^^42^ These dialysis-related factors may affect cognitive test performance independently of bone turnover marker levels and may have introduced measurement variability or residual confounding that could not be fully accounted for in this cross-sectional study. The timing of cognitive assessment, such as before dialysis, after dialysis, or on non-dialysis days, should be standardized in future studies, and dialysis-related hemodynamic or cerebral perfusion parameters should be incorporated.

This study has some additional limitations. First, the cross-sectional design precludes causal inference, and reverse causation remains possible. Second, bone turnover markers are surrogate measures and do not directly define underlying bone histology; total ALP is not bone-specific and may reflect extra-skeletal sources despite adjustment for hepatobiliary parameters and comorbid liver disease. In addition, gold-standard assessments of bone turnover, such as bone histomorphometry, were not available. This limits the ability to distinguish true high-turnover bone disease from marker-specific systemic pathways. Third, although cognitive impairment was identified, a definitive clinical diagnosis of dementia could not be established. Nevertheless, two complementary cognitive assessments (MoCA and MMSE) were used to enhance reliability. Fourth, residual confounding is possible, including factors not fully captured in our dataset, such as depressive symptoms and physical activity. Fifth, although missingness was low for most variables, smoking status had a higher proportion of missing data and was handled as an “unknown” category, whereas other analyses were conducted as complete-case analyses. Sixth, information on bisphosphonate use was not available in our dataset. Although bisphosphonates are generally used with caution in patients undergoing hemodialysis, unmeasured use of these agents may have influenced bone turnover marker levels and could not be fully accounted for. Finally, our cohort comprised older patients from seven centers in two prefectures in Japan. This may limit generalizability to other settings and populations.

Our findings suggest several directions for future research. First, prospective studies are needed to evaluate whether routinely measured bone turnover markers, particularly BAP and total ALP, can improve risk stratification for cognitive decline in patients undergoing hemodialysis. Such work should assess incremental predictive value beyond established risk factors and consider multivariable models incorporating bone turnover markers alongside clinical, nutritional, and inflammatory parameters. Second, mechanistic studies are warranted to clarify whether bone metabolism and ALP-related pathways are physiologically linked to cognitive dysfunction in patients undergoing hemodialysis, including integration with imaging markers of cerebrovascular disease and vascular or valvular calcification. Where feasible, studies incorporating more definitive assessments of bone turnover, such as histomorphometry from bone biopsy, may help disentangle whether the observed associations reflect true bone turnover or marker-specific systemic pathways. Finally, if a causal link is supported, interventional studies targeting CKD–MBD and high bone turnover may be justified to determine whether modifying bone turnover can mitigate cognitive decline.

In this exploratory cross-sectional study of older patients undergoing hemodialysis, BAP was inversely associated with cognitive performance, whereas TRACP-5b was not. Total ALP showed an inverse association that weakened after extensive adjustment. These findings suggest that formation-related bone turnover markers may be relevant for risk stratification of cognitive impairment in patients undergoing hemodialysis. Future longitudinal and mechanistic studies are needed to determine whether these routinely measured markers can aid risk stratification and to clarify underlying pathways.

## Supplementary Material

Supplementary_Material_7_3_ziag124

## Data Availability

The data underlying this article cannot be shared publicly due to concerns that they contain information that could compromise the privacy of research participants. The data will be shared on reasonable request to the corresponding author.
