# Supplementary material for: Bone turnover markers associated with cognitive dysfunction in patients undergoing hemodialysis: a cross-sectional study
Source: JBMR Plus. 2026 Aug 5;10(10):ziag124. doi: 10.1093/jbmrpl/ziag124 (PMC13537456; doi:10.1093/jbmrpl/ziag124)
Supplement: Supplementary file 1 [file supplementary_material_7_3_ziag124.docx]

**SUPPLEMENTARY MATERIAL**

**Supplementary Table 1.** Multivariable logistic regression analyses of the association between bone turnover markers and cognitive dysfunction (Montreal Cognitive Assessment ≤25 and ≤22)

|  | **Montreal Cognitive Assessment ≤25** | | **Montreal Cognitive Assessment ≤22** | |
| --- | --- | --- | --- | --- |
|  | **Odds ratio (95% CI)** | **P value** | **Odds ratio (95% CI)** | **P value** |
| **Log BAP** |  |  |  |  |
| Unadjusted | 1.32 (0.82, 2.12) | 0.251 | 1.49 (0.92, 2.40) | 0.102 |
| Model 1 | 1.24 (0.74, 2.06) | 0.408 | 1.93 (1.13, 3.29) | 0.015 |
| **Log total ALP** |  |  |  |  |
| Unadjusted | 1.35 (0.77, 2.36) | 0.293 | 2.03 (1.19, 3.46) | 0.009 |
| Model 1 | 1.21 (0.69, 2.15) | 0.492 | 2.39 (1.33, 4.27) | 0.003 |
| **Log TRACP-5b** |  |  |  |  |
| Unadjusted | 1.21 (0.89, 1.64) | 0.230 | 0.92 (0.66, 1.28) | 0.605 |
| Model 1 | 1.16 (0.82, 1.64) | 0.410 | 1.09 (0.76, 1.56) | 0.656 |

CI, confidence interval; BAP, bone-specific alkaline phosphatase; ALP, alkaline phosphatase; TRACP-5b, tartrate-resistant acid phosphatase 5b.

Bone turnover markers were natural log–transformed. Facility was adjusted for using fixed effects (indicator variables).

**Unadjusted:** marker only.

**Model 1:** Adjusted for age, sex, and facility.

**Supplementary Table 2.** Multivariable linear regression analyses of the association between bone turnover markers and MoCA domain scores

| **MoCA domain** | **Marker** | **Unadjusted β (95% CI)** | **P value** | **Model 1 β (95% CI)** | **P value** |
| --- | --- | --- | --- | --- | --- |
| **Visuospatial/ executive** | Log BAP | -0.12 (-0.36, 0.11) | 0.302 | -0.26 (-0.50, -0.03) | 0.028 |
|  | Log total ALP | -0.19 (-0.46, 0.07) | 0.149 | -0.23 (-0.48, 0.03) | 0.085 |
|  | Log TRACP-5b | 0.02 (-0.14, 0.18) | 0.801 | -0.14 (-0.31, 0.03) | 0.104 |
| **Naming** | Log BAP | 0.03 (-0.04, 0.09) | 0.424 | -0.00 (-0.06, 0.05) | 0.880 |
|  | Log total ALP | 0.02 (-0.04, 0.08) | 0.495 | 0.02 (-0.04, 0.08) | 0.550 |
|  | Log TRACP-5b | 0.07 (0.01, 0.12) | 0.024 | 0.04 (-0.01, 0.09) | 0.133 |
| **Attention** | Log BAP | -0.16 (-0.39, 0.08) | 0.198 | -0.12 (-0.35, 0.12) | 0.319 |
|  | Log total ALP | -0.17 (-0.44, 0.09) | 0.194 | -0.12 (-0.38, 0.13) | 0.334 |
|  | Log TRACP-5b | -0.09 (-0.24, 0.06) | 0.256 | -0.07 (-0.23, 0.09) | 0.368 |
| **Language** | Log BAP | -0.24 (-0.44, -0.05) | 0.013 | -0.27 (-0.46, -0.08) | 0.006 |
|  | Log total ALP | -0.26 (-0.47, -0.05) | 0.015 | -0.23 (-0.44, -0.02) | 0.029 |
|  | Log TRACP-5b | -0.13 (-0.25, -0.01) | 0.041 | -0.16 (-0.29, -0.03) | 0.014 |
| **Abstraction** | Log BAP | -0.08 (-0.19, 0.02) | 0.120 | -0.12 (-0.22, -0.01) | 0.030 |
|  | Log total ALP | -0.21 (-0.34, -0.08) | 0.002 | -0.21 (-0.34, -0.07) | 0.003 |
|  | Log TRACP-5b | 0.05 (-0.04, 0.14) | 0.285 | -0.00 (-0.08, 0.08) | 0.998 |
| **Delayed recall** | Log BAP | -0.18 (-0.52, 0.16) | 0.298 | -0.15 (-0.49, 0.18) | 0.363 |
|  | Log total ALP | -0.25 (-0.64, 0.14) | 0.205 | -0.23 (-0.61, 0.15) | 0.233 |
|  | Log TRACP-5b | -0.20 (-0.42, 0.02) | 0.080 | -0.16 (-0.39, 0.07) | 0.175 |
| **Orientation** | Log BAP | -0.15 (-0.30, 0.00) | 0.053 | -0.15 (-0.30, 0.00) | 0.056 |
|  | Log total ALP | -0.22 (-0.39, -0.04) | 0.017 | -0.19 (-0.37, -0.02) | 0.031 |
|  | Log TRACP-5b | -0.04 (-0.11, 0.03) | 0.243 | -0.05 (-0.12, 0.03) | 0.213 |

MoCA, Montreal Cognitive Assessment; CI, confidence interval; BAP, bone-specific alkaline phosphatase; ALP, alkaline phosphatase; TRACP-5b, tartrate-resistant acid phosphatase 5b. Bone turnover markers were natural log-transformed. Facility was adjusted for using fixed effects (indicator variables). Domain-level analyses were exploratory.

**Unadjusted:** marker only. **Model 1:** Adjusted for age, sex, and facility.

**Supplementary Table 3.** Clinical determinants of MoCA score

| **Variable** | **Unadjusted β (95% CI)** | **P value** | **Fully adjusted model β (95% CI)** | **P value** | **Std. β** |
| --- | --- | --- | --- | --- | --- |
| Log BAP | -0.79 (-1.58, -0.001) | 0.050 | -1.03 (-1.90, -0.16) | 0.021 | -0.13 |
| Male sex | 0.07 (-0.73, 0.87) | 0.856 | 0.64 (-0.37, 1.66) | 0.213 | 0.09 |
| Age, years | -0.17 (-0.22, -0.12) | <0.001 | -0.12 (-0.18, -0.07) | <0.001 | -0.23 |
| Education ≤12 years | -0.55 (-1.25, 0.16) | 0.127 | -0.04 (-0.79, 0.72) | 0.928 | -0.00 |
| Log dialysis vintage | 0.14 (-0.17, 0.45) | 0.369 | -0.02 (-0.40, 0.37) | 0.931 | -0.01 |
| Pre-dialysis systolic blood pressure | 0.00 (-0.01, 0.02) | 0.757 | 0.01 (-0.01, 0.03) | 0.259 | 0.06 |
| Diabetes mellitus | -0.26 (-0.99, 0.46) | 0.472 | -0.51 (-1.29, 0.26) | 0.195 | -0.07 |
| Cardiovascular disease | 0.01 (-0.74, 0.76) | 0.971 | -0.11 (-0.93, 0.71) | 0.798 | -0.01 |
| History of stroke | -1.86 (-3.02, -0.70) | 0.002 | -1.54 (-2.74, -0.34) | 0.012 | -0.16 |
| Chronic hepatitis | 0.19 (-1.50, 1.88) | 0.826 | -0.04 (-1.88, 1.80) | 0.966 | -0.00 |
| Hepatocellular carcinoma | 1.66 (-0.50, 3.82) | 0.132 | 1.40 (-0.76, 3.56) | 0.204 | 0.05 |
| Phosphate binder use | 0.51 (-0.27, 1.29) | 0.200 | -0.12 (-0.93, 0.69) | 0.771 | -0.02 |
| Vitamin D receptor activator use | 0.14 (-0.71, 0.99) | 0.744 | -0.23 (-1.08, 0.61) | 0.587 | -0.03 |
| Calcimimetic use | 0.54 (-0.18, 1.26) | 0.140 | 0.07 (-0.71, 0.85) | 0.867 | 0.01 |
| Denosumab use | -1.07 (-2.43, 0.29) | 0.123 | 0.65 (-0.99, 2.30) | 0.434 | 0.04 |
| Systemic glucocorticoid use | -1.08 (-2.74, 0.57) | 0.199 | -0.56 (-2.59, 1.47) | 0.590 | -0.03 |
| Urea nitrogen | 0.04 (0.01, 0.07) | 0.003 | 0.02 (-0.01, 0.05) | 0.234 | 0.07 |
| Single-pool Kt/V | -0.28 (-1.58, 1.01) | 0.670 | 0.97 (-0.43, 2.36) | 0.174 | 0.08 |
| Albumin | 1.52 (0.40, 2.64) | 0.008 | 1.09 (-0.35, 2.53) | 0.137 | 0.10 |
| Body mass index | 0.10 (0.01, 0.18) | 0.037 | 0.04 (-0.07, 0.15) | 0.480 | 0.04 |
| Hemoglobin | 0.28 (-0.02, 0.58) | 0.070 | 0.24 (-0.11, 0.58) | 0.181 | 0.08 |
| Log C-reactive protein | -0.21 (-0.43, 0.00) | 0.054 | -0.06 (-0.31, 0.19) | 0.635 | -0.03 |
| Log γ-glutamyl transferase | -0.12 (-0.62, 0.39) | 0.647 | 0.29 (-0.23, 0.80) | 0.272 | 0.06 |
| Calcium | 0.46 (-0.07, 0.99) | 0.089 | 0.37 (-0.30, 1.05) | 0.278 | 0.06 |
| Phosphate | 0.36 (0.07, 0.65) | 0.015 | -0.00 (-0.35, 0.34) | 0.989 | -0.00 |
| Log intact parathyroid hormone | 0.32 (-0.16, 0.81) | 0.188 | 0.12 (-0.38, 0.63) | 0.628 | 0.03 |
| Log soluble α-klotho | 0.18 (-0.55, 0.92) | 0.630 | 0.13 (-0.64, 0.89) | 0.743 | 0.02 |
| Log 25-hydroxyvitamin D | -0.68 (-1.37, 0.00) | 0.049 | -0.61 (-1.41, 0.18) | 0.127 | -0.09 |
| Log fibroblast growth factor 23 | 0.26 (-0.03, 0.54) | 0.080 | -0.19 (-0.50, 0.13) | 0.244 | -0.08 |
| MoCA, Montreal Cognitive Assessment; CI, confidence interval; Std. β, standardized beta coefficient; BAP, bone-specific alkaline phosphatase; Kt/V, single-pool urea clearance. Log-transformed variables were natural log-transformed. Univariable analyses were performed using available cases among participants with available BAP and each variable. The fully adjusted model included all variables listed in the table, as well as facility and smoking status. Facility was adjusted for using fixed effects, and smoking status was modeled as a three-level categorical variable (yes/no/unknown). Standardized β values were obtained from the fully adjusted complete-case model and are shown to compare the relative magnitude of associations; they should be interpreted cautiously for binary variables. | | | | | |
